# The relationship between organizational justice and bullying behaviors among nurses: the role of nurse managers’ caring behaviors

**DOI:** 10.1186/s12912-024-02134-1

**Published:** 2024-07-23

**Authors:** Ebtsam Aly Abou Hashish, Sharifa Alsayed, Hend Abdu Alnajjar, Siti Awa Abu Bakar

**Affiliations:** 1 College of Nursing - Jeddah, King Saud Bin Abdul-Aziz University for Health Sciences, King Abdulaziz Medical City, National Guard Health Affairs, Mail Code 6565, P.O.Box.9515, Jeddah, 21423 Saudi Arabia; 2 King Abdullah International Medical Research Center, Jeddah, Saudi Arabia; 3 Faculty of Nursing, Alexandria University, Alexandria, Egypt

**Keywords:** Organizational justice, Bullying behaviors, Nurses, Nurse managers, Managerial caring, Mediating effect

## Abstract

**Background:**

Organizational justice is pivotal in fostering a fair and supportive workplace culture, which strengthens the connections between managers and nurses, among nurses themselves, and ultimately, between nurses and their patients. Assessing the perceived levels of organizational justice and managerial behaviors can identify key areas for improving nurses’ commitment and loyalty, while simultaneously reducing incidents of workplace bullying.

**Purpose:**

This study aims to investigate how bedside nurses perceive organizational justice, nurse managers’ caring behaviors, and their exposure to workplace bullying. Additionally, it seeks to explore the relationship between organizational justice, nurse managers’ caring behaviors, and nurses’ perceived exposure to workplace bullying.

**Methods:**

A descriptive-correlational study was conducted in the inpatient care unit of a Saudi hospital. A convenience sample of 256 nurses participated, completing the Organizational Justice Questionnaire (OJQ), the Caring Factor Survey: Caring of the Manager (CFS-CM), and the Negative Acts Questionnaire-Revised (NAQ-R). The collected data were analyzed using descriptive statistics and regression analysis.

**Results:**

Descriptive statistics revealed moderate levels of perceived organizational justice and managerial caring behaviors among nurses, alongside low reported exposure to workplace bullying. Significant correlations were found among the studied variables, indicating that higher perceived organizational justice was associated with higher managerial caring and lower workplace bullying (*p* < 0.05). Mediation analysis demonstrated a significant indirect effect of organizational justice on workplace bullying through the mediating role of nurse managers’ caring behaviors (a×b = -0.0652, *p* < 0.001). Furthermore, the direct effect of organizational justice on workplace bullying remained significant even when accounting for the mediator (c = -0.5509, *p* < 0.001).

**Conclusion:**

This study highlights the vital role of organizational justice and managerial caring in cultivating a positive work environment and mitigating workplace bullying. Implementing clear policies and procedures while promoting fairness and equality in resource allocation, decision-making processes, and interactions are essential strategies for fostering positive attitudes and work behaviors among nurses.

**Supplementary Information:**

The online version contains supplementary material available at 10.1186/s12912-024-02134-1.

## Introduction

In contemporary healthcare environments, organizations encounter substantial challenges in retaining nursing staff and fostering justice, as emphasized by numerous researchers [1, 2]. Nurses increasingly advocate for fair treatment, recognizing organizational justice as pivotal to their workplace contentment [1]. Organizational justice (OJ), a cornerstone of success, profoundly influences nurses’ morale and efficiency [3]. Research consistently illustrates the adverse consequences of perceived injustice, including emotional distress and workplace bullying (WPB) [2, 4, 5]. This study investigates how perceived justice among employees could predict workplace bullying, emphasizing the importance of fostering fairness and empathy in healthcare organizations through the caring behaviors of nurse managers. Understanding nurses’ perspectives on organizational justice (OJ) and managerial caring (MC) is paramount for identifying areas to bolster commitment and reduce workplace bullying (WPB) incidents [2, 6]. Furthermore, the research endeavors to elucidate the impact of nurse managers’ caring behaviors on WPB among nurses. Identifying effective interventions to tackle WPB is critical, given the current ambiguity surrounding prevention measures [7]. Thus, illuminating the role of OJ and MC behaviors in mitigating WPB is essential for devising targeted interventions and fostering supportive healthcare environments.

Limited research in Saudi Arabia has explored the association between nurses’ perceptions of OJ, nurse managers’ caring behaviors, and their encounters with WPB within hospital settings. Consequently, this study aims to address this knowledge gap. Investigating this relationship and gauging the levels of OJ and MC could prove instrumental in identifying workplace variables that are amenable to change. Such insights could empower organizational leaders to effectively tackle dysfunctional behaviors and significantly influence nurses’ commitment to their workplace [8].

## Literature review and hypotheses development

The current study investigates the perceived levels of OJ, MC, and WPB, as well as the intricate relationship among variables in healthcare settings. Drawing from an extensive literature review, the hypotheses are developed, integrating both theoretical frameworks and empirical evidence. Foundational to this investigation are seminal works by Colquitt [9], which highlight the significance of fairness perceptions in organizational contexts. Complementing this perspective, the Watson Theory of Human Caring enriches the conceptual framework, highlighting the importance of compassionate interactions and interpersonal connections in fostering a culture of care within organizations. Furthermore, insights from Nelson and Watson’s examination of nurse managers’ caring behaviors emphasize the critical role of MC in cultivating a supportive work environment [10].

**Fig. 1:**
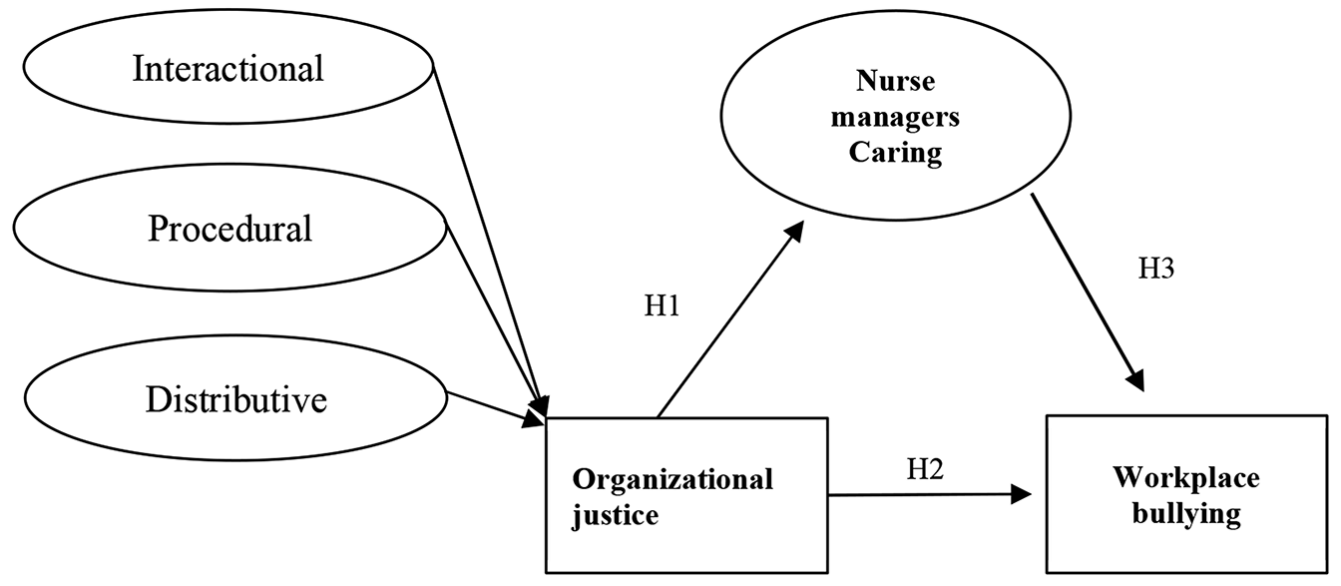
Proposed study framework

Additionally, the conceptualization of WPB in nursing, as outlined by Einarsen et al. [11], provides valuable insights into the manifestations and consequences of such behaviors in the healthcare sector. The study framework, depicted in Fig. 1, synthesizes these theoretical underpinnings to offer a holistic understanding of the factors influencing WPB. Underpinning this research is the social exchange theory. The Social Exchange Theory [12] illuminates the reciprocal relationships and resource exchanges that occur within social contexts. Through this interdisciplinary lens, the study aims to provide valuable insights into how OJ and MC can effectively mitigate WPB, ultimately enhancing the well-being and productivity of healthcare professionals in Saudi Arabia.

Other theories, like the Affective Events Theory and the Fairness Heuristic Theory, provide theoretical support for this study. For instance, the Affective Events Theory (AET) posits that emotional reactions triggered by workplace events significantly influence employees’ attitudes and behaviors. By incorporating AET into the conceptual framework, this study acknowledges the profound impact of affective responses on perceptions of fairness and support, thereby providing a nuanced understanding of how these factors contribute to workplace dynamics in the healthcare sector. Furthermore, AET suggests that positive affective experiences, such as compassionate interactions and supportive managerial behaviors, can mitigate the adverse effects of WPB, fostering a more conducive work environment for healthcare professionals [13].

Likewise, within the conceptual framework of the study, the fairness heuristic theory provides valuable insights. According to this theory, individuals rely on simple heuristic rules to assess fairness in complex social situations. By applying the fairness heuristic lens, the study explores healthcare professionals’ cognitive processes in judging the fairness of organizational practices and interpersonal interactions. Additionally, the theory underscores the importance of perceived fairness in shaping employees’ attitudes and behaviors, highlighting the potential role of fairness perceptions in mitigating workplace bullying [14].

### Organizational justice and nurse managers’ caring behaviors

Organizational justice (OJ) pertains to nurses’ assessments of whether they have been treated fairly within their organization and how these assessments influence various work-related factors [1]. It encompasses nurses’ perceptions of the fairness of workplace practices, interactions, and outcomes. These perceptions can significantly affect their productivity, commitment, and, ultimately, the organization’s success [3]. Researchers have developed various models to measure organizational justice. Colquitt [9] proposed a well-known model that identifies three dimensions or components of OJ: interactional, procedural, and distributive justice. Interactional justice refers to the degree to which workers are treated properly and respectfully within an institution [15–17]. It pertains to the manner in which superiors treat individuals with courtesy, honesty, and respect [17]. In the context of this study, interactional justice concerns the relationships between nurse managers and nurses, including how nurse managers treat nurses in daily activities and decision-making, as well as how they communicate procedures and approaches for distributing outcomes such as evaluation results, salary increases, and incentives [9].

Procedural Justice relates to the consistent application of regulations, laws, and company policies, as well as the procedure for performance evaluation. It is also related to the perceived fairness and appropriateness of individuals in the decision-making process in organizations [15, 16, 18]. This includes how clear and reliable results are implemented, the capacity to voice opinions during the process, ethical and impartial decision-making or lack of prejudice, and correctness [15]. Therefore, procedural justice emphasizes the impartiality of the policies and procedures through which outcomes are determined [16]. Distributive Justice involves the fair distribution of resources and workload [1, 9]. The principle of distributive justice is drawn from Equity Theory [19] which illustrates how individuals compare their results (rewards) to inputs (knowledge, skills, and abilities) relative to other people [16–20]. These dimensions will serve as metrics for assessing perceived OJ in the current study through using the Organizational Justice Questionnaire (OJQ).

The interaction between caring behaviors of nurse managers and OJ forms a strong foundation for effective nursing leadership in complex healthcare settings [21]. Research indicates that OJ plays a key role in predicting positive outcomes in the workplace [22]. To explain these positive effects, the social exchange theory (SET) [12] offers a helpful framework. SET suggests that when employees engage in mutually beneficial relationships, they develop positive attitudes and behaviors, beyond just economic gains [23]. Therefore, SET helps to understand how justice (OJ), including fairness in interactions, procedures, and distribution, relates to the caring behavior of managers. It has been delineated that organizations promoting fair treatment, respectful interactions, ethical decision-making, and fair compensation create an environment where employees feel comfortable sharing information and feedback. This fosters positive behaviors in both managers and staff [22]. Moreover, studies show that fair treatment within organizations leads to managers demonstrating caring behavior [1, 21]. Based on this, Therefore, we hypothesize:

*H1: Organizational justice positively correlates with nurse managers’ caring behavior.*

### Nurse managers caring behaviors and workplace bullying

Caring is fundamental to the nursing profession, representing its core attribute [24]. Watson’s theory of human caring delineates ten Caritas processes that elucidate nurses’ and nurse managers’ ways of understanding and being. These processes facilitate care by (1) demonstrating loving kindness; (2) engaging in problem-solving and decision-making; (3) instilling faith and hope; (4) facilitating teaching and learning; (5) respecting spiritual beliefs and practices; (6) adopting a holistic approach; (7) fostering trusting relationships; (8) creating healing environments; (9) encouraging emotional expression; and (10) acknowledging miracles (reflective of a belief in a higher power). Nurse managers utilize these Caritas processes to embody managerial caring behaviors, which are viewed as therapeutic and healing interventions due to their social nature and emphasis on honoring the integrity and individuality of each person [21–25]. In this study, nurse managers’ caring behaviors are operationally defined as nurses’ ratings on the Caring Factor Survey: Manager’s Caring (CFS-CM) developed by Nelson and Watson [10]. This widely recognized instrument measures nurses’ perceptions of nurse managers’ caring behaviors, drawing on Caritas concepts central to Watson’s theory of human caring [21, 24, 25]. Previous research underscores the importance of nurse managers’ caring behaviors and supportive leadership behaviors in mitigating negative workplace phenomena such as workplace bullying [6, 26].

Workplace bullying is a prevalent issue worldwide. In response, the American Nurses Association Code of Ethics for Nurses [27] emphasizes the importance of fostering work environments free from bullying, harassment, and threatening behaviors. According to the Workplace Bullying Institute [28], workplace bullying is characterized by the repetitive mistreatment of one or more individuals (the targets) by one or more perpetrators. This mistreatment can take various forms, including verbal abuse, threats, intimidation, humiliation (both verbal and nonverbal), and actions that obstruct work progress, such as sabotage. Bullies typically target individuals who lack support or are unable to defend themselves against aggression. The victims of bullying often view themselves as victims, subjected to deliberate and prolonged negative treatment, and feel helpless to defend themselves [28].

Einarsen et al. [11] proposed three factors to operationally define exposure to workplace bullying: personal bullying, work-related bullying, and physically intimidating bullying. Personal bullying encompasses behaviors like yelling, spreading rumors, making derogatory jokes, and delivering offensive criticism. Work-related bullying includes actions such as setting unrealistic deadlines, assigning unmanageable workloads, withholding crucial work-related information, disregarding opinions, and coercing individuals to forfeit their rights. Physically intimidating bullying involves exposure to actual or potential acts of violence or physical abuse [11].

The Joint Commission (JC) [29] has highlighted that workplace bullying is prevalent in environments characterized by stress and hostility, compounded by factors such as unclear anti-bullying policies, ineffective communication, unfair treatment among peers, a lack of supportive atmosphere, hierarchical structures, organizational changes, and deficient leadership skills. According to the JC [29] approximately 50% of nurses experience bullying and/or disruptive behavior, with over 90% reporting witnessing such conduct in their workplaces. Unfortunately, nurses often perceive nurse-on-nurse bullying as an accepted aspect of their profession, particularly among new or inexperienced nurses, leading to the term “nurses eat their young” [28]. In the Saudi context, the study by Alhassan et al. [30] investigated workplace bullying and violence within the healthcare sector surveying 7398 healthcare workers (HCWs) in Saudi Arabia. They found that approximately 26.6% of respondents had experienced workplace bullying within the past year. Notably, the primary perpetrators were managers or supervisors (26.3%), followed by other staff members (23.1%) and patients (20.1%). Regrettably, only 11.3% of these incidents underwent formal investigation. They reported that the reluctance to report bullying incidents stemmed from a perceived futility in doing so and the fear of potential negative repercussions. Although actions were taken against 87.1% of the offenders, these primarily consisted of verbal warnings (44.8%), with only 1.7% facing more severe consequences such as discontinuation of medical care. Moreover, the study revealed dissatisfaction among bullied individuals with the actions taken by their supervisors [30].

Further analysis revealed that nurses comprised a significant portion (38.7%) of the non-bullied HCWs compared to those who reported exposure (36.5%). Additionally, Saudi national HCWs were notably more susceptible to bullying compared to their non-Saudi counterparts [30]. This result aligns with previous research by Harthi et al. and Benardes et al., which also found female nurses to be the primary victims of workplace bullying [31, 32]. Nurses’ vulnerability to bullying may stem from their perceived lower status within the healthcare hierarchy and insufficient training on how to deal with such situations. Moreover, the fear of retaliation from higher authorities may deter nurses from speaking up about bullying incidents, fearing job loss or salary reduction.

Scholars have noted that workplace bullying has a significant impact on the nursing field, adversely affecting nurses’ mental well-being and performance, and resulting in adverse physical and psychological consequences [2, 33]. Studies have documented various negative outcomes associated with workplace bullying, including diminished job satisfaction and engagement, heightened turnover rates, and the development of psychological disorders like moral distress [34, 35]. Prolonged exposure to unaddressed workplace bullying can even lead to conditions such as posttraumatic stress disorder and self-destructive behavior [7]. These findings underscore the urgent need for comprehensive interventions to address workplace bullying and create safer and more caring environments for healthcare workers, particularly nurses, in Saudi Arabia [30]. Previous research also indicates that nurses’ perceptions of managerial support significantly influence their perception of workplace bullying [6]. Therefore, we hypothesize:

*Hypothesis H2: Nurse managers’ caring behaviors negatively correlates with bullying behaviors among nurses.*

### Organizational justice, managerial caring, and workplace bullying: exploring the interplay

Organizational justice (OJ) is a cornerstone in understanding how employees perceive fairness within their workplace, exerting a profound influence on their behavior and attitudes [17, 18]. Extensive research has established OJ as a critical predictor of employee behavior. Fair treatment from organizations correlates strongly with heightened levels of commitment, trust, satisfaction, and ethical conduct among nurses [9]. This conducive environment fosters effective collaboration with managers and colleagues and encourages employees to engage in extra-role behaviors, as they perceive organizational policies and procedures to be ethical and equitable [16]. Conversely, instances of organizational injustice are linked with an increased susceptibility to workplace aggression and bullying. Victims of bullying often attribute their experiences to feelings of unfair treatment, underscoring the direct association between perceptions of organizational injustice and the prevalence of bullying [11].

Moreover, prior research further substantiates the pivotal role of managerial caring as a significant predictor of reduced bullying behaviors [6]. Managerial support plays a pivotal role in fostering positive work behaviors, as committed employees tend to go above and beyond their job requirements. Managerial caring serves as potential mediators between organizational justice and employee outcomes. Support from managers encourages heightened employee engagement, leading to positive job behaviors and a reduction in negative work behaviors, such as bullying [36, 37]. Without managerial support, employees may not produce sustainable outcomes, and any achieved success may be temporary and eventually dissipate [38]. Therefore, the effective implementation of OJ is anticipated to correlate with heightened MC, thereby resulting in decreased occurrences of WPB.

Supporting these perspectives, this study is grounded in the assumptions of social exchange theory (SET) and Watson’s theory of human caring. Social exchange theory (SET) posits that relationships are built on reciprocal exchanges of resources, where positive managerial actions, such as fairness and support, encourage employees to reciprocate with higher commitment and positive behaviors [12]. Watson’s theory of human caring emphasizes the importance of creating a caring environment where the humanistic aspects of nursing are valued, promoting trust, empathy, and support [24, 25]. These theoretical frameworks explore the indirect influence of organizational justice on bullying behavior through managerial support and nurse managers’ caring behaviors. Specifically, the study examines how organizational justice—perceived fairness in organizational processes and interactions—influences nurse caring behaviors, characterized by empathy, support, and ethical care [9]. These caring behaviors, in turn, mitigate workplace bullying by fostering a respectful and supportive work environment [11]. By understanding the role of justice and caring in creating a supportive work environment, hospital and nurse managers can better appreciate how managerial support acts as a crucial resource to sustain positive employee outcomes, such as increased job satisfaction, and reduce negative behaviors, such as bullying. Therefore, we hypothesize:

*Hypothesis H3: Nurse managers’ caring behaviors mediate the relationship between organizational justice and workplace bullying behaviors among nurses.*

### Significance of the study

This study’s significance lies in its exploration of workplace dynamics within healthcare organizations. Nurses, who are pivotal to patient care and organizational productivity, often face bullying from a variety of sources, including administrators, supervisors, peers, and even patients and their families [39]. However, it remains unclear whether managerial support and caring behaviors can effectively counteract such incidents [6]. This gap in understanding is crucial, as a lack of managerial intervention may perpetuate a culture of bullying, eroding nurses’ trust and confidence [6, 40].

Previous studies have examined the impact of organizational justice on various outcomes and behaviors among nurses, such as workplace deviance and bullying [1, 2, 41]. Additionally, research has explored the relationship between workplace bullying and deviant work behaviors [42], as well as the connection between nurse managers’ caring behaviors and workplace bullying [40]. As a result, organizational justice is an important core value within organizations [43]. These studies have identified contributing factors to workplace bullying, including a culture that tolerates bullying, resource inadequacy, excessive workloads, power imbalances, and ineffective leadership and management. Moreover, role ambiguity, high levels of stress, a lack of autonomy, and organizational injustice can all contribute to workplace bullying. However, none of these studies have simultaneously assessed the influence of organizational justice on managerial caring and bullying behaviors. Furthermore, the generalizability of previous research findings on organizational justice to other countries and to nurses specifically remains underexplored [5].

The study’s significance extends beyond academia, given the significant implications of workplace bullying on employee well-being and organizational efficiency, especially in Saudi Arabia [30]. With its unique cultural and professional landscape, understanding the prevalence and impact of workplace bullying is crucial. Therefore, investigating the relationship between organizational justice, managerial caring behaviors, and workplace bullying is essential, given the documented links between unfair practices, stress, and negative workplace behaviors. Ultimately, this study holds practical implications for nurses, nurse managers, healthcare administrators, and policymakers. It enhances our understanding of workplace dynamics and provides insights to inform interventions and policies aimed at fostering healthier and more productive work environments, particularly in Saudi Arabia.

#### Aim of the study

The present study aims to investigate how nurses perceive their organizational justice, nurse managers’ caring behaviors, and exposure to workplace bullying in their hospital. Further, to investigate the relationship among these variables.

## Methods

### Study Design and setting

Researchers conducted a descriptive-correlational study at the inpatient care unit of King Khalid Hospital (KKH) in Jeddah, Saudi Arabia.

#### Study participants

The study aimed to include all nurses employed in inpatient care units at KKH, totaling 350 individuals. Eligible participants were registered nurses with a minimum of six months of work experience who provided written consent. The study excluded newly hired nurses and student nurses. The Raosoft Sample Size calculator, using specified parameters such as a population size of 350, a margin error of 5, a confidence interval of 95%, and a significance level of 0.05, recommended a minimum sample size of 184 for nurses. However, in order to achieve the desired sample size, we distributed 300 questionnaires to nurses, resulting in a convenient sample of 256 nurses who completed and returned the questionnaires.

### Study Measurement Tools (Instruments)

#### Nurses’ demographic and work characteristics

This tool includes various variables such as age, gender, nationality, educational attainment, department of employment, years of experience, nurse-to-patient ratio, weekly work hours, shift schedule, and participation in extra shifts.

#### Organizational justice questionnaire (OJQ)

To assess perceived organizational justice, Colquitt [9] introduced the OJQ, comprising 20 items distributed across three dimensions: interactional justice (9 items), procedural justice (6 items), and distributive justice (5 items), which collectively determine overall organizational justice. Sample item is “the hospital administration takes work decisions in an unbiased manner with everyone.” Respondents rated their experiences of fair interactions, procedures, and outcomes on a 5-point Likert scale ranging from 1 (to a small extent) to 5 (to a great extent). Total scores range from 20 to 100, with higher mean scores indicating greater perceived organizational justice.

#### The caring factor survey: manager’s caring (CFS-CM)

The CFS-CM developed by Nelson and Watson [10] consists of 10 items, each corresponding to one of Watson’s ten caritas concepts. For example, the statement “I observe my manager treating employees with loving kindness every day” aligns with the caritas concept of “practice of loving kindness and spiritual regard.” Nurses rate their nurse managers’ caring behaviors on a 5-point Likert response scale from “5” (strongly agree) to “1” (strongly disagree). Scores for caritas concepts range from 10 to 50, with higher scores indicating a greater perception of caring behavior among nurse managers.

#### Negative acts Questionnaire-revised (NAQ-R)

Einarsen et al. [11] developed the NAQ-R to measure perceptions of the frequency of workplace bullying behaviors. It consists of 22 items categorized into three bullying factors: person-related bullying (12 items), work-related bullying (7 items), and physically intimidating bullying (3 items). Sample item is “Intimidating behavior such as finger-pointing, invasion of personal space, shoving, blocking/barring the way.” Responses indicate the frequency of exposure to bullying factors on a 5-point Likert scale, ranging from “5 = daily” to “1 = never.” According to Einarsen et al. [11], being exposed to negative acts at least twice a week for six months meets the criteria for bullying. Total scores range from 22 to 110, with higher scores indicating greater nurses’ exposure to bullying behaviors. These instruments are available for public research use.

### Validity and reliability

Considering that all participants were university graduates with a high proficiency in English (a requirement for employment at the hospital), the study instruments were administered in English. The internal consistency and reliability of the instruments were assessed using Cronbach’s alpha correlation coefficient. The results demonstrated high reliability, with coefficients of 0.823, 0.811, and 0.847 for the OJQ, CFS-CM, and NAQ-R scales, respectively. A significance level of *p* ≤ 0.05 was applied. Content validity was determined through evaluation by the researchers and expert academic members in the relevant field, and a pilot study was conducted with 5% of nurses, with no subsequent modifications made.

### Data collection

After receiving approval from the Institutional Review Board (IRB) and obtaining consent from the hospital’s director and nurse managers, the researchers prepared the study questionnaire in both paper-based and electronic formats. Each nursing station within the units had a QR code for the electronic version available. Nurses were individually approached by the researchers via email and WhatsApp to inquire about their preferred method for completing the questionnaire (paper-based or electronic). Following informed consent, the researchers clarified the study’s objectives and provided any necessary guidance to the participants. Data collection occurred over a four-month period, from September 2023 to December 2023.

### Data analysis

The researchers utilized the Statistical Package for the Social Sciences (SPSS) version 25 for data coding and statistical analysis. Data cleaning procedures were conducted to address any missing data, and the normal distribution of the data was confirmed through the Shapiro-Wilk test. Frequency and percentages presented demographic characteristics, while the mean and standard deviation summarized the data using descriptive statistics. Correlation and regression analyses were employed to identify significant predictors (e.g., OJ) and outcome variables (e.g., NAQ-R). The R^2^ change was evaluated using the F-test and analysis of variance (ANOVAs). To explore the mediating effect of nurse managers’ caring behavior on workplace bullying behavior, the Hayes process macro model was applied. A significance level of *p* ≤ 0.05 was utilized for all analyses.

### Ethical considerations

Approvals for the study were obtained from the College of Nursing-Jeddah (CONJ), the hospital’s director, and the King Abdullah International Medical Research Center (KAIMRC) Institutional Review Board (IRB) under reference [NRJ23J/142/06], ensuring compliance with ethical guidelines and regulations governing human research. Before participating, all nurses provided written informed consent after receiving a comprehensive explanation of the study’s objectives, potential risks and benefits, and their voluntary right to withdraw at any stage. The researchers prioritized safeguarding data privacy, ensuring confidentiality, and maintaining participant anonymity.

## Results

### Demographic and professional characteristics

A total of 256 nurses took part in the study, with the majority being female (*n*238, 93.0%). Among the participants, most were non-Saudi nurses (*n* = 160, 60.2%) and fell within the age range of 30 to 40 years old (*n* = 190, 74.2%). Nurses were evenly spread across various patient care units, with representation ranging from 10.9 to 25.0%. The highest proportion of nurses (*n* = 226, 88.3%) held a bachelor’s degree, and a significant number (*n* = 170, 66.4%) had between 6 and 10 years of professional experience. Detailed demographic and professional characteristics are provided in Table 1.

**Table 1:** Distribution of the studied nurses according to demographic and work characteristics (*N* = 256)

Demographic and work Characteristics	No.	%
**Gender**		
Male	18	7.0
Female	238	93.0
**Nationality**		
Saudi	96	37.5
Non-Saudi	160	62.5
**Age category**		
20 - <30 years	44	17.2
30 - <40 years	190	74.2
40 - <50 years	14	5.5
≥ 50 years	8	3.1
**Educational level**		
Bachelor level	226	88.3
Institute diploma	22	8.6
Master	8	3.1
**Primary work area or unit in this hospital?**		
Emergency department	34	13.3
Intensive care unit (any type) unit	64	25.0
Medical	40	15.6
Obstetrics	28	10.9
Oncology	50	19.5
Surgical	40	15.6
**Years of experience in your current specialty or profession?**		
Less than 1 year	16	6.3
1 to 5 years	42	16.4
6 to 10 years	170	66.4
11 to 15 years	12	4.7
16 to 20 years	8	3.1
21 years or more	8	3.1

### Descriptive analysis of overall organizational justice, nurse managers’ caring behaviors and workplace bullying among the studied nurses

Table 2 presents the descriptive statistics for the variables under study. The overall mean percentage score and standard deviation (SD) for organizational justice were 58.26 ± 18.49, indicating that nurses perceived a moderate level of organizational justice within their hospital. This trend was consistent across the three domains of organizational justice. Specifically, nurses perceived distributive justice slightly higher (mean = 59.10 ± 18.97) compared to procedural justice (mean = 57.55 ± 27.34) and interactional justice (mean = 58.63 ± 18.45).

Additionally, Table 2 illustrates the overall mean and standard deviation of nurse managers’ caring behaviors as 73.89 ± 19.91, indicating that nurses perceived their first-line nurse managers as moderately caring. The majority of nurses agreed that their managers treated employees with kindness (86.8%), supported their individual spiritual beliefs (73.4%), and established a helpful and trusting relationship with them. Furthermore, many nurses reported that their managers taught them in a manner they could understand (72.7%). However, only about half of the nurses (52.4%) agreed that their managers were accepting and supportive of their beliefs regarding a higher power, suggesting a need for more care and support in this aspect. For further details on caring behaviors, refer to supplementary Table 1. On the other hand, nurses reported a lower level of workplace bullying overall (mean = 21.20 ± 20.48), as well as lower scores for person-related, work-related, and physically intimidating bullying. Specifically, work-related bullying was reported as the lowest (mean = 1.77 ± 0.86), indicating that nurses perceived this type of bullying to be less prevalent in their workplace.

**Table 2:** Descriptive analysis of overall organizational justice, nurse managers’ caring behaviors and workplace bullying among the studied nurses (*N* = 256)

Variables	Average Score	% Score
	Mean ± SD.	Mean ± SD.
Organizational Justice	3.33 ± 0.74	58.26 ± 18.49
Interactional justice	3.30 ± 1.09	57.55 ± 27.34
Procedural justice	3.35 ± 0.74	58.63 ± 18.45
Distributive justice	3.36 ± 0.76	59.10 ± 18.97
**Nurse managers’ caring behaviors**	**3.96 ± 0.80**	**73.89 ± 19.91**
**Workplace bullying**	**1.85 ± 0.82**	**21.20 ± 20.48**
Person-related bullying	1.86 ± 0.85	21.53 ± 21.13
Work-related bullying	1.77 ± 0.86	19.36 ± 21.50
Physically intimidating bullying	1.97 ± 0.73	24.15 ± 18.37

SD:Standard deviation

### The correlation and regression among organizational justice, nurse managers’ caring behaviors and workplace bullying with the mediating role of managerial caring

Table 3 shows that perceived organizational justice has a positive and significant correlation with managerial caring (*r* = 0.299, *p* < 0.001), and both organizational justice and nurse managers’ caring behaviors have negative correlations with workplace bullying (*r* = -0.556, *p* < 0.001; *r* = -0.345, *p* < 0.001, respectively). The correlation matrix also reveals significant relationships among various dimensions of organizational justice with managerial caring and workplace bullying. Interactional Justice demonstrates a strong positive correlation with managerial caring (*r* = 0.799, *p* < 0.001) and a strong negative correlation with workplace bullying (*r* = -0.556, *p* < 0.001). Procedural Justice shows a moderate positive correlation with managerial caring (*r* = 0.299, *p* < 0.001) and a stronger negative correlation with workplace bullying (*r* = -0.445, *p* < 0.001) compared to Distributive Justice, which exhibits a weaker positive correlation with managerial caring (*r* = 0.253, *p* < 0.001) and a moderate negative correlation with workplace bullying (*r* = -0.357, *p* < 0.001).

**Fig. 2:**
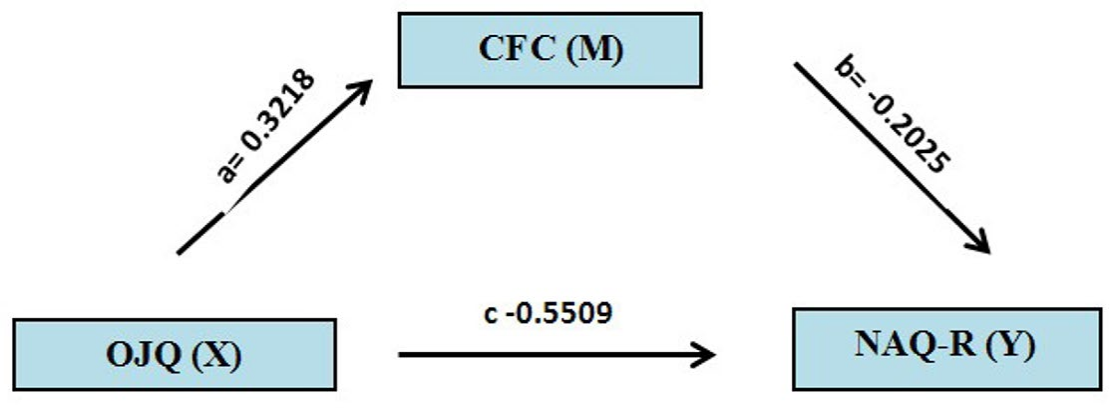
The relationship among organizational justice, managerial caring and workplace bullying

The multivariate regression analysis in Table 4 further highlighted the significant impact of overall organizational justice (OJ) and nurse managers’ caring behaviors (CFS-CM) on workplace bullying behaviors among nurses (OJ: 𝛽 = -0.551, *p* < 0.001; CFS-CM: 𝛽 = -0.202, *p* < 0.001). Additionally, the mediation analysis showed a significant indirect effect of organizational justice on workplace bullying through the mediating role of nurse managers’ caring behaviors (axb = -0.0652, *p* < 0.001). The direct effect of organizational justice on workplace bullying remained significant even in the presence of the mediator (c = -0.5509, *p* < 0.001). See Table 5; Fig. 2.

**Table 3:** Correlation matrix among organizational justice and it dimensions, managerial caring and workplace bullying

OJQ Organizational Justice & dimensions		CFS-CM Managerial Caring	NAQ-*R* Workplace bullying
**Organizational Justice**	**r**	*r* = 0.299	-0.556
	**p**	< 0.001^*^	< 0.001^*^
Interactional Justice	**r**	0.799	-0.556
	**p**	< 0.001^*^	< 0.001^*^
Distributive Justice	**r**	0.253	-0.357
	**p**	< 0.001^*^	< 0.001^*^
Procedural Justice	**r**	0.299	-0.445
	**p**	< 0.001^*^	< 0.001^*^

r: Pearson coefficient *: Statistically significant at *p* ≤ 0.05

**Table 4:** Multivariate regression analysis for the parameters affecting workplace bullying behaviors among nurses

Variables	B	t	*p*	(LL – UL 95%C.I)
**OJQ**	-0.551	9.325^*^	< 0.001^*^	-0.667 – -0.435
**CFS-CM** ^a^	-0.202	3.692^*^	< 0.001^*^	-0.310 – -0.094

r: Pearson coefficient ^a^ r between NAQ-R and CFS-CM =-0.345^*^

r between OJQ and CFS-CM = 0.299*

B: Unstandardized Coefficients, t: t-test of significance C.I: Confidence interval, LL: Lower limit. UL: Upper Limit *: Statistically significant at *p* ≤ 0.05

**Table 5:** Mediation analysis of the relationship between Organizational Justice and Workplace bullying

Relationship	Total Effect	Direct Effect	Indirect Effect	95% CI		t–statistics	Conclusion
				LL	UL		
**OJQ–> CFS- >** **NAQ-R**	-0.6161 (< 0.001^*^)	-0.5509 (< 0.001)	-0.0652 (< 0.001^*^)	-0.1253	-0.0163	-2.329 (> 1.96) (sig < 0.05)	Partial Mediation

*: Statistically significant at *p* ≤ 0.05

### The analysis of the difference in the perceived studied variables according to nurses’ demographic and work characteristics variables

Furthermore, the analysis of the difference in the perceived studied variables according to nurses’ demographic and work characteristics variables showed some significant difference according to nationality, years of experience, nurse- to patient ratio, working shift, number of hours worked per week, and work extra shifts during the month (see Table 6). For instance, Saudi nurses showed a higher mean for OJ than other nurses (t = 2.320, *p* = 0.021). Nurses with 21 years or more of experience showed a higher mean of MC (f = 7.247, *p* < 0.001) and a lower mean of WPB (f = 3.559, *p* = 0.004). Nurses who were assigned to 1–4/5 patients reported a higher mean of both OJ (f = 4.565, *p* = 0.004) and MC (f = 19.134, *p* < 0.001). Nurses who worked 36 h per week showed the highest mean for both OJ and MC (*p* < 0.001), while these worked 60 h and more reported higher WPB than others (f = 14.902, < 0.001). Nurses who worked day shifts showed a higher OJ than those who worked rotating shifts (t = 5.076, *p* < 0.001), while nurses working rotating shifts showed a higher mean for MC (t = 12.033, *p* < 0.001). Nurses who did not work extra shifts during the month had a higher perception of OJ (t = 3.226, *p* = 0.002) and a lower perception of WPB (t = 6.254, *p* < 0.001).

**Table 6:** Difference in perceived variables according to nurses’ demographic and work characteristics (*N* = 256)

Demographic and work Characteristics	OJ	MC	WPB
**Nationality**			
Saudi	3.47 ± 0.76	4.04 ± 0.76	1.87 ± 0.84
Non-Saudi	3.25 ± 0.72	3.90 ± 0.82	1.83 ± 0.81
**t (p)**	**2.320 (0.021** ^*^ **)**	1.376 (0.170)	0.354 (0.724)
**Years of experience in your current specialty or profession?**			
Less than 1 year	3.39 ± 1.15	4.34 ± 0.49	1.78 ± 0.89
1 to 5 years	3.29 ± 0.84	4.29 ± 0.87	1.80 ± 0.87
6 to 10 years	3.36 ± 0.63	3.87 ± 0.75	1.89 ± 0.76
11 to 15 years	3.28 ± 1.03	3.52 ± 0.33	1.46 ± 0.76
16 to 20 years	2.65 ± 1.07	3.10 ± 1.15	2.65 ± 1.38
21 years or more	3.63 ± 0.05	4.68 ± 0.60	1.15 ± 0.02
**F (p)**	1.739 (0.126)	**7.247 (< 0.001** ^*^ **)**	**3.559 (0.004** ^*^ **)**
**Nurse- to Patient Ratio**			
1–2	3.07 ± 0.95	4.16 ± 0.82	1.87 ± 0.89
1–3	3.27 ± 0.92	4.15 ± 1.06	1.93 ± 1.0
1–4/5	3.64 ± 0.46	4.38 ± 0.48	1.75 ± 0.85
1-More than 5 patients	3.30 ± 0.58	3.55 ± 0.46	1.83 ± 0.63
**F (p)**	**4.565 (0.004** ^*^ **)**	**19.134** ^*^ **(< 0.001** ^*^ **)**	0.448 (0.719)
**Working shift**:			
Day	3.67 ± 0.47	3.25 ± 0.37	1.94 ± 0.84
Rotating between day and night	3.24 ± 0.77	4.14 ± 0.77	1.82 ± 0.81
**t (p)**	**5.076** ^*^ **(< 0.001** ^*^ **)**	**12.033** ^*^ **(< 0.001** ^*^ **)**	**0.889 (0.375)**
**Number of hours worked per week and working shift**			
36	3.60 ± 0.0	4.90 ± 0.0	1.55 ± 0.0
48	3.39 ± 0.69	3.98 ± 0.73	1.77 ± 0.74
60	2.79 ± 1.0	3.51 ± 1.12	2.63 ± 1.12
**F (p)**	**8.490** ^*^ **(< 0.001** ^*^ **)**	**8.981** **(< 0.001** ^*^ **)**	**14.902 (< 0.001** ^*^ **)**
**Working extra shifts during the month**			
Yes	3.28 ± 0.74	3.95 ± 0.85	1.93 ± 0.83
No	3.67 ± 0.65	4.02 ± 0.14	1.33 ± 0.45
**t (p)**	**3.226 (0.002** ^*^ **)**	1.262 (0.208)	**6.254**(< 0.001^*^)

SD: Standard deviation t: Student t-test F: F for One way ANOVA test.

p: p value for comparing between the studied categories *: Statistically significant at *p* ≤ 0.05.

## Discussion

Hospitals and nurse managers face a significant challenge in establishing and upholding a supportive work environment characterized by fairness, justice, and caring managers. This correlational and descriptive investigation extends previous research by exploring the influence of organizational justice (OJ) on workplace bullying behaviors (WPB) among nurses and the mediating role of managerial caring behaviors. Our findings reveal significant correlations among dimensions of organizational justice, managerial caring behaviors, and workplace bullying. Perceived organizational justice has a positive correlation with managerial caring behaviors, indicating that fair treatment within organizations is associated with more supportive managerial actions, supporting Hypothesis 1. Recent studies corroborate these findings. For instance, Colquitt et al. [44] and Zarish et al. [45] consistently report that organizational justice positively influences managerial behaviors that demonstrate care and support for employees. This aligns with our results, emphasizing the role of fairness perceptions in shaping managerial caring.

Likewise, the study reveals positive correlations between specific dimensions of organizational justice and other perceived variables. Specifically, interactional, procedural, and distributive justice show positive correlations with managerial caring, indicating that perceptions of fairness in interpersonal treatment and organizational procedures are associated with more supportive managerial behaviors. Conversely, these dimensions exhibit significant negative correlations with workplace bullying, with interactional justice showing the highest correlation and distributive justice the lowest. Recent research by Fouquereau et al. [46] highlights the pivotal role of interactional justice in fostering supportive managerial behaviors and positive employee outcomes. Additionally, Lotfi-Bejestani et al. [47] underscore the importance of procedural justice in enhancing perceived fairness and reducing workplace conflicts. Our findings are consistent with this perspective, suggesting that while distributive justice positively correlates with managerial caring, its influence on reducing workplace bullying may be comparatively limited compared to interactional and procedural justice dimensions. Recently, Solinas-Saunders et al. [48] found significant positive correlations between all three dimensions of organizational justice (interactional, procedural, and distributive) and supervisory and management trust. Hence, the current study emphasizes the critical role of organizational justice dimensions, particularly interactional and procedural justice, in shaping both managerial relationships and the prevalence of workplace bullying. Organizations that prioritize fairness in interpersonal treatment and transparent procedural practices are likely to cultivate more supportive managerial environments and effectively reduce incidents of workplace bullying.

The regression analysis results highlight a negative association between OJ and WPB, indicating that OJ may serve as a predictor of WPB occurrences. Furthermore, the study finds that OJ has a significant direct impact on workplace bullying, even when a mediator is present. As a result, nurse managers’ caring behaviors emerge as a partial mediator in the association between OJ and WPB, lending support to Hypothesis 2. A descriptive analysis of variable levels and mean scores supports these results, revealing a moderate perception of OJ and nurse managers’ caring behaviors, but a lower perception of WPB behaviors. This suggests a link between fewer bullying incidents and higher perceptions of fairness and supportive managerial caring behaviors.

This result could be clarified by the idea that workers who see their organization as fair are more likely to view their managers as empathetic and supportive. This implies that organizations that place importance on equity and impartiality are more likely to cultivate positive relationships between managers and employees. Furthermore, individuals who perceive fair and dignified treatment from their peers are less susceptible to encountering hostility and mistreatment [1, 2]. Mohamed et al. [2] observed a notable negative correlation between organizational justice and workplace bullying. Similarly, Neall et al. [41] emphasized a moderate association between organizational justice and workplace bullying. On the other hand, perceptions of injustice within the organization, such as disparities in treatment or unjust policies, are associated with increased instances of workplace bullying. These findings underscore the pivotal role of organizational justice in nurturing favorable work environments [1, 44, 45, 49]. By prioritizing equity and fairness, organizations can foster compassionate managerial conduct and alleviate adverse workplace behaviors. This viewpoint is consistent with the research of Abou Hashish and Khatab [6].

Moreover, the findings suggest that caring behaviors demonstrated by nurse managers can partially mediate the influence of OJ on WPB, supporting Hypothesis 3. This study fills a gap in the literature by providing empirical evidence and theoretical insights into the variables associated with WPB. Specifically, nurses who perceive organizational justice and experience caring behaviors from their nurse managers are less likely to engage in workplace bullying. This mediation effect aligns with Social Exchange Theory, wherein nurses reciprocate positive attitudes and behaviors when they feel supported and valued by their managers and organization, thus reducing instances of workplace bullying [50]. Authentic empathy, concern for well-being, and addressing injustice. By intervening and providing support to employees experiencing injustice, managers can create a safer and more positive work environment, effectively mitigating workplace bullying. This perspective resonates with Mohammad et al. [51], who emphasize the importance of nurse managers’ role modeling. Nurse managers serve as trustworthy leaders, exhibiting self-awareness, open-mindedness, adherence to moral principles, and creating a supportive and ethical work environment conducive to nurses’ autonomy and growth.

The current study findings are consistent with previous research. For example, Abou Hashish and Khatab [6] demonstrated that nurse managers’ caring behaviors were significantly correlated with nurses’ reduced perceptions of WPW. Similarly, Bortoluzzi et al. [26] identified negative associations between supportive leadership behaviors and WPB, highlighting the role of supportive leadership in mitigating bullying occurrences. Additionally, Elliethey et al. [52] established negative correlations and predictive links between work ethics and negative work behaviors, indicating that supportive work environments result in fewer instances of negative behaviors among nurses. Numerous studies have underscored the significance of fostering empowering work environments, promoting strong work ethics, nurturing supportive relationships between managers and nurses, and implementing fair performance management systems and reasonable incentive structures. By instituting flexible work systems and cultivating a positive organizational culture that prioritizes ongoing support, learning, and development, hospitals can contribute to fostering a positive work attitude among nurses [1, 45, 51–53]. This supportive approach can effectively address negative work behaviors, enhance nurses’ retention and satisfaction, and ultimately enhance the quality of patient care [52].

Alsharah [54] conducted a study in Saudi ministries and found that employees perceived their working environment with justice, indicating that their perception of fairness, equity, and distributive justice within the organization strongly influences their engagement with their work. Interactional justice followed closely, highlighting the importance of fair and consistent interpersonal treatment. In contrast, procedural justice was perceived as having the least impact among organizational justice dimensions, suggesting that while the fairness of organizational processes is relevant, it is overshadowed by perceptions of distributive justice and interpersonal interactions [54].

*Regarding the descriptive level of the studied variables*, the present study indicates that nurses perceive moderate levels of OJ, with distributive justice rating slightly higher than procedural and interactional justice. The hospital’s fair resource allocation, transparent decision-making processes, and supportive work environment contribute to nurses feeling fairly treated and valued. Various factors inherent to the hospital environment, such as hospital policies, resource allocation, workload distribution, and communication practices, could contribute to the moderate levels of organizational justice observed among nurses. Previous research conducted in the same study setting confirms that the findings likely reflect the specific organizational dynamics and culture present within the studied hospital [53, 55].

Recent studies support the notion that establishing fair resource and reward distribution, transparent decision-making procedures, and fostering positive work environments can enhance nurses’ perceptions of justice. Abou Hashish [1] observed moderate levels of organizational justice and underscored the importance of cultivating fairness and respect in the workplace to improve employee well-being and perceptions of justice. Similarly, TopbaÅŸ et al. [56] identified a significant correlation between nurses’ perceptions of organizational justice, job satisfaction, and burnout levels, advocating for institutions to adopt fair policies and encourage personal development among nurses. Chin et al. [57] examined how perceptions of justice influence nurse retention, highlighting workplace justice as a protective factor against nurses leaving their profession.

The emphasis on distributive justice may stem from its concrete characteristics, allowing individuals to assess the fairness of outcomes more easily than procedural or interactional justice. This reasoning is supported by recent justice research [58, 59]. Also, this finding aligns with existing research and theoretical frameworks on organizational justice. Prior studies consistently show that distributive justice tends to receive higher average scores compared to procedural and interactional justice [60]. Lee and Rhee [60] found that organizational justice significantly contributes to organizational sustainability, with distributive justice having a particularly strong impact on employee motivation. However, they recommend that managers focus not only on enhancing distributive justice but also on promoting procedural and interactional justice within the organization. Likewise, Liu et al. [61] pointed out that organizational justice, especially distributive justice, notably impacts the inclination for inter-organizational cooperation and positive conduct among employees. Furthermore, Zahednezhad et al. [49] discovered that both distributive and interactional justice were pivotal in diminishing nurses’ intentions to exit the profession by positively influencing their job satisfaction. Both studies recommended implementing fair performance appraisal systems and enhancing workplace autonomy to discourage nurses from leaving the nursing profession.

Our investigation revealed that nurses generally perceived their immediate nurse managers as demonstrating a moderate level of managerial care. Specifically, the majority of nurses concurred that their managers showed kindness towards employees and respected their spiritual beliefs, indicating a favorable view of their supervisors’ caring demeanor. This observation is consistent with the notion that humanistic care is pivotal in managing clinical nurses. Additionally, Yousef et al. [62] stressed the influential role of nurse leaders as exemplars, which can influence the professional values of clinical nurses. Through the transmission of humanistic care, nurses perceive the caring attitude of nurse managers and subsequently extend it to patients and colleagues, thereby enhancing the quality of nursing care [63]. Our study’s results align with those of Liao et al. [63] who investigated the extent of nurse managers’ caring behaviors and identified factors that hindered or facilitated the implementation of humanistic care. They reported positive caring behaviors exhibited by nurse managers and emphasized the reciprocal nature of caring, highlighting the significance of establishing trust to enable clinical nurses to embrace and benefit from the provided care. Similarly, Abou Hashish and Khatab documented that nurse managers demonstrated a moderate level of caring, as perceived by nurses, underscoring the importance of caring leadership and the substantial role of nurse manager caring behaviors in fostering a positive and supportive work environment [6].

Despite this outcome, it is worth noting that only half of the participating nurses reported that their nurse managers were supportive and accepting of their beliefs about a higher power, which allows for the possibility of personal and professional growth. Additionally, 45.3% of nurses remained neutral in their responses, suggesting a varied landscape of perceptions among the participants. This variation likely indicates the influence of cultural and personal differences, as well as nationality and religious beliefs. In our study, all Saudi nurses and nurse managers shared the same Muslim religious background and language. In contrast, about half of the nurse managers were non-Saudis, and some were non-Muslims. This diversity may have an impact on how people perceive and communicate support for and acceptance of beliefs about a higher power. In cultures where religious beliefs are integral to personal and professional identities, the alignment or misalignment of these beliefs between nurses and their managers may have a significant impact on perceptions of managerial support. Previous research has highlighted the importance of religious and cultural congruence in workplace relationships. For instance, the study by Almutairi [64] pointed to the challenges faced by non-Saudi and non-Muslim healthcare workers in predominantly Muslim countries like Saudi Arabia, suggesting that cultural and religious differences can influence workplace dynamics and perceptions of managerial support. Alotaibi et al. [65] showed that religion and spiritual beliefs can enhance nurses’ job satisfaction. In this instance, Swihart et al. [66] emphasized the importance of cultural and religious competence in clinical practice. Strategies to move health professionals and systems towards these goals include providing cultural competence training and developing policies and procedures that decrease barriers to providing culturally competent patient care.

Moreover, the current study revealed a low prevalence of workplace bullying (21.20%) among nurses, with work-related bullying showing particularly low levels. Various factors, including the hospital environment, research methods, and contextual or cultural influences, could explain this result. The supportive environment of the hospital under study may have contributed to our study’s lack of significant differences in bullying perceptions between Saudi and non-Saudi nurses. The supportive work environment of the hospital under study, combined with organizations’ growing awareness of the prevalence and harmful effects of workplace bullying, has spurred increased efforts to prevent it. Previous studies, which emphasized the positive work culture within this hospital [53, 55], could support these results. Additionally, fostering a positive work culture and encouraging effective communication among employees are crucial elements that can potentially reduce the incidence of workplace bullying, as highlighted by Smith et al. [59] and Goh et al. [67]. Moreover, the chosen research methodology, particularly the reliance on self-reporting to evaluate incidents of workplace bullying, might influence the recorded low rates. There is a possibility that nurses may have given incorrect responses, potentially downplaying instances of bullying to manage their image and avoid potential consequences. This observation is consistent with the findings of Jönsson and Muhonen [68] and Abou Hashish and Khatab [6], who proposed that employees may hesitate to participate in surveys addressing sensitive topics like workplace bullying. Employees may perceive that voicing their concerns would not lead to any changes, or they may fear adverse effects on their employment situation if they were to disclose such incidents.

Furthermore, Alhassan et al. [30] highlighted that approximately 26.6% of healthcare workers in Saudi Arabia have experienced workplace bullying, with managers or supervisors being the primary perpetrators. Notably, nurses comprised a significant portion (38.7%) of the non-bullied healthcare workers compared to those who reported exposure (36.5%). Additionally, Saudi national healthcare workers were notably more susceptible to bullying compared to their non-Saudi counterparts. There is a notable reluctance to report such incidents, primarily due to perceived futility and fear of negative repercussions. This context helps to understand the low-level perception of workplace bullying observed in our study, suggesting that while bullying may not be prevalent, nurses, especially non-Saudis, may be hesitant to report it [30].

Although our study revealed a lower occurrence of workplace bullying, it is essential to acknowledge that the prevalence and forms of such behavior can vary across different healthcare settings and regions. Extensive research consistently highlights the frequent encounters nurses have with workplace bullying and harassment. For instance, Abou Hashish and Khatab [6] revealed that approximately 66.67% of nurses experienced bullying, with work-related bullying being the most common, followed by physical intimidation and interpersonal bullying. Similarly, Trépanier et al. [69] found that up to 40% of nurses faced bullying, while Houck and Colbert [70] reported prevalence rates ranging from 26 to 77%. These statistics underscore the significant impact of bullying on healthcare. Kang and Lee [71] also emphasized the widespread nature of workplace bullying in nursing, particularly affecting newly graduated nurses. The high bullying prevalence rate reported among nurses warrants an urgent need for nurse leaders to address this issue. This highlights the importance of exploring both individual and organizational strategies to prevent workplace bullying among nurses. Therefore, despite our study’s findings of a relatively lower level of workplace bullying, it is essential to recognize the broader context and ongoing challenges in addressing this issue within the nursing profession. The complexity of workplace bullying reporting and the influence of cultural and personal factors must be acknowledged. Future studies should indeed consider investigating these aspects in greater detail to provide a more comprehensive understanding of workplace bullying in Saudi hospitals.

Finally, the analysis of differences in perceived studied variables according to nurses’ demographic and work characteristics showed significant differences based on nationality, years of experience, nurse-to-patient ratio, working shift, number of hours worked per week, and work extra shifts during the month. Saudi nurses had a higher average score for organizational justice compared to other nurses. This may be attributed to cultural factors and a sense of national pride and identity within the workplace, which could foster a perception of fairness and equity among local employees. Similarly, Al-Aameri [72] found that cultural factors significantly influence perceptions of organizational justice among employees in Saudi Arabia. More experienced nurses perceived more managerial caring. This could be because experienced nurses have had more time to build relationships with their managers and may be more adept at navigating the organizational culture, thereby perceiving greater managerial support and care. This finding concurs with Duffield et al. [73], who noted that experienced nurses often have stronger relationships with management, leading to higher perceived managerial support.

Workload-related variables appear to influence perceived organizational justice and managerial caring. Nurses with a moderate number of patients, lower working hours, and those who did not work extra shifts reported higher perceptions of organizational justice and managerial caring. This suggests that manageable workloads allow for better quality interactions with management and more perceived fairness and care. Excessive workloads, on the other hand, might strain these interactions, reducing perceptions of organizational justice and managerial support. These results are similar to the findings of Wynendaele et al. [74] and Alsayed [75], who reported that manageable workloads are associated with higher perceptions of fairness and support.

Moreover, nurses working 60 h or more reported higher levels of workplace bullying. This finding concurs with the notion that excessive working hours can lead to stress and burnout, creating an environment where bullying is more likely to occur [10, 67]. The high levels of stress and fatigue associated with long working hours can reduce resilience and increase vulnerability to negative behaviors [76]. Also, the current study revealed that experienced nurses and those who did not work extra shifts reported lower levels of workplace bullying. Experienced nurses may have developed better coping mechanisms and strategies to deal with workplace stress and conflict, resulting in fewer workplace bullying incidents. Similarly, Goh et al. [67] found that age and length of experience were negatively associated with workplace bullying, while nurses with less locus of control or poor compliance with social norms were at greater risk [77]. Additionally, avoiding extra shifts might help reduce overall stress and fatigue, contributing to a more positive work environment and lowering the incidence of bullying. This finding is consistent with the conclusions of Karatuna et al. [77] and Trépanier et al. [69], who reported that better job characteristics, higher quality interpersonal relationships, people-centric leadership styles, and a positive organizational culture promoting staff empowerment, distributive justice, and zero tolerance for bullying were associated with reduced workplace bullying.

These findings underscore the importance of managing workloads and fostering supportive relationships within healthcare settings to enhance organizational justice and managerial caring. Specifically, ensuring manageable nurse-to-patient ratios, reasonable working hours, and minimizing the need for extra shifts can contribute to higher perceptions of fairness and support among nurses. Furthermore, experienced nurses can serve as mentors, helping newer staff navigate the workplace and build stronger, more supportive relationships with management [52–55, 78].

### Strengths and limitations

This study presents several strengths that significantly enrich the existing literature. Firstly, it adds a distinctive contribution by examining the relationship between organizational justice, managerial caring, and workplace bullying among nurses, filling a void in prior research. The findings are supported by empirical evidence, providing solid backing for the proposed associations and presenting valuable theoretical insights through the application of Social Exchange Theory and Theory of Human Caring to elucidate the mediating role of managerial caring. Nonetheless, it is crucial to recognize certain limitations. The study’s cross-sectional design restricts its capacity to establish causal relationships between variables. Additionally, relying on self-report measures, particularly for assessing workplace bullying, might introduce response bias. The outcomes reflect nurses’ perceptions of organizational justice and nurse managers’ caring behaviors rather than an objective evaluation. Considering that individuals may interpret and anticipate fair treatment and care differently, the findings likely represent the subjective experiences and perspectives of the nurses. Moreover, confining the study to a single hospital setting limits the generalizability of the findings to diverse healthcare contexts. It is possible that the study did not account for all potential confounding variables that influenced the observed correlations. Despite these limitations, the study’s outcomes hold the potential to enhance our understanding of nursing dynamics and make significant contributions to the broader field of hospital management. It is essential to address these limitations in future research endeavors.

## Conclusion

In summary, this study emphasizes the essential role of nursing leadership in nurturing a positive work atmosphere and enhancing nurses’ job performance. It indicates that higher levels of organizational justice and managerial caring correlate with fewer instances of workplace bullying. By prioritizing fairness, equity, and supportive leadership approaches, healthcare institutions can enhance nurses’ welfare and patient care outcomes. Furthermore, the study demonstrates that managerial caring partially mediates the relationship between organizational justice and workplace bullying, emphasizing the importance of supportive leadership in mitigating negative workplace conduct.

### Implications of the study

#### Implications for practice and nursing management

The results of this study have significant implications for practice in healthcare settings. It is crucial for healthcare organizations to invest in training programs for nurse managers focused on fostering supportive work environments and effectively addressing workplace bullying. Developing clear policies and procedures to address workplace bullying, with an emphasis on zero-tolerance and accessible reporting mechanisms, is imperative. Leaders should prioritize fairness and equality in resource allocation, decision-making processes, and interactions to cultivate perceptions of organizational justice among nurses.

#### Implications for future research

Future research should consider employing longitudinal designs to explore the long-term effects of organizational justice, managerial caring, and workplace bullying among nurses, thereby enhancing the applicability of the findings. Investigating these relationships across diverse healthcare settings could shed light on contextual factors influencing workplace dynamics. Organizational justice and managerial caring intervention studies are needed to reduce workplace bullying, improve nurses’ well-being, and enhance job satisfaction. Furthermore, future research should address study limitations by employing a variety of data collection sources and methods, including both qualitative and quantitative approaches, as well as diverse and representative samples. Furthermore, expanding the scope of the investigation to include various individual-related and organizational variables could provide a more comprehensive understanding of workplace bullying. Exploring the relationship between organizational justice, managerial caring, and positive outcomes such as organizational citizenship behavior and job satisfaction could offer additional insights into the implications of caring leadership and supportive work environments.

Moreover, future research could benefit from employing a triangulation approach to validate these findings. This approach would combine quantitative data with qualitative insights from interviews or focus groups to deepen our understanding of the phenomenon of workplace bullying. Additionally, further studies should explore the comparative perceptions and effects of workplace bullying among diverse nursing populations, specifically between foreign and local nurses. This would help elucidate how cultural backgrounds influence the perception, reporting, and coping mechanisms related to workplace bullying.

### Electronic supplementary material

Below is the link to the electronic supplementary material.

Supplementary Material 1

## Data Availability

All the data generated or analyzed during this study are included in this published article and supplementary file.

## Abbreviations

AET: Affective Events Theory
ANOVA: Analysis of variance
CFS-CM: Caring Factor Survey: Caring of the Manager
CONJ: College of Nursing-Jeddah
IRB: Institutional Review Board
JC: Joint Commission
KAIMRC: King Abdullah International Medical Research Center
MC: Managerial Caring
NAQ-R: Negative Acts Questionnaire-Revised
OJ: Organizational Justice
SET: Social Exchange Theory
OJQ: Organizational Justice Questionnaire
SD: Standard deviation
WPB: Workplace bullying behaviors
