# Supplementary material for: The relationship between organizational justice and bullying behaviors among nurses: the role of nurse managers’ caring behaviors
Source: BMC Nurs. 2024 Jul 23;23:503. doi: 10.1186/s12912-024-02134-1 (PMC11265478; doi:10.1186/s12912-024-02134-1)
Supplement: Supplementary file 1 — Supplementary Material 1 [file 12912_2024_2134_MOESM1_ESM.docx]

**Supplementary Table 1. Distribution of perceived managerial caring
items among the studied nurses (N = 256)**

| **Managerial caring** | **Strongly Disagree** | | **Disagree** | | **Neutral** | | **Agree** | | **Strongly Agree** | | **Agreement** |
| --- | --- | --- | --- | --- | --- | --- | --- | --- | --- | --- | --- |
|  | **No.** | **%** | **No.** | **%** | **No.** | **%** | **No.** | **%** | **No.** | **%** | **%** |
| **Every day I am here I see my manager treats employees with loving kindness.** | 0 | 0.0 | 8 | 3.1 | 26 | 10.2 | 144 | 56.3 | 78 | 30.5 | 86.8 |
| **My manager is good at creative problem solving to meet my individual needs and requests.** | 4 | 1.6 | 2 | .8 | 76 | 29.7 | 106 | 41.4 | 68 | 26.6 | 68 |
| **The manager of my unit/department helps instills hope and respects my belief system.** | 2 | 0.8 | 4 | 1.6 | 78 | 30.5 | 94 | 36.7 | 78 | 30.5 | 67.2 |
| **When my manager teaches me something new, s/he teaches me in a way that I can understand.** | 0 | 0.0 | 4 | 1.6 | 66 | 25.8 | 106 | 41.4 | 80 | 31.3 | 72.7 |
| **The manager of my unit/department encourages me to practice my own individual spiritual beliefs as part of my self caring.** | 2 | 0.8 | 4 | 1.6 | 62 | 24.2 | 106 | 41.4 | 82 | 32.0 | 73.4 |
| **The manager of my unit/department responds to me as a whole person, helping to take care of all my needs and concerns.** | 4 | 1.6 | 4 | 1.6 | 72 | 28.1 | 96 | 37.5 | 80 | 31.3 | 68.8 |
| **The manager of my unit/department has established a helping and trusting relationship with me during my time here on this unit/department.** | 4 | 1.6 | 4 | 1.6 | 62 | 24.2 | 106 | 41.4 | 80 | 31.3 | 72.7 |
| **The manager of my unit/department creates a healing environment in our unit/department that recognizes the connection between body, mind, and spirit.** | 4 | 1.6 | 8 | 3.1 | 70 | 27.3 | 96 | 37.5 | 78 | 30.5 | 68 |
| **I feel like I can talk openly and honestly with** | 6 | 2.3 | 4 | 1.6 | 86 | 33.6 | 90 | 35.2 | 70 | 27.3 | 62.5 |
| **The manager of my unit/department is accepting and supportive of my beliefs regarding a higher power, which allows for the possibility of me to ‘grow.’** | 0 | 0.0 | 6 | 2.3 | 116 | 45.3 | 54 | 21.1 | 80 | 31.3 | 52.4 |
|  |  |  |  |  |  |  |  |  |  |  |  |
